# Refractive prediction of four different intraocular lens calculation formulas compared between new swept source optical coherence tomography and partial coherence interferometry

**DOI:** 10.1371/journal.pone.0251152

**Published:** 2021-05-04

**Authors:** Mi Yeon Song, Sung Rae Noh, Kook Young Kim

**Affiliations:** Kim’s Eye Hospital, Seoul, Korea; University of Toronto, CANADA

## Abstract

**Purpose:**

To compare the biometry and prediction of postoperative refractive outcomes of four different formulae (Haigis, SRK/T, Holladay1, Barrett Universal II) obtained by swept-source optical coherence tomography (SS-OCT) biometers and partial coherence interferometry (PCI; IOLMaster ver 5.4).

**Methods:**

We compared the biometric values of SS-OCT (ANTERION, Heidelberg Engineering Inc., Heidelberg, Germany) and PCI (IOLMaster, Carl Zeiss Meditec, Jena, Germany). Predictive errors calculated using four different formulae (Haigis, SRKT, Holladay1, Barrett Universal II) were compared at 1 month after cataract surgery.

**Results:**

The mean preoperative axial length (AL) showed no statistically significant difference between SS-OCT and PCI (SS-OCT: 23.78 ± 0.12 mm and PCI: 23.77 ± 0.12 mm). The mean anterior chamber depth (ACD) was 3.30 ± 0.04 mm for SS-OCT and 3.23 ± 0.04 mm for PCI, which was significantly different between the two techniques. The mean corneal curvature also differed significantly between the two techniques. The difference in mean arithmetic prediction error was significant in the Haigis, SRKT, and Holladay1 formulae. The difference in mean absolute prediction error was significant in all four formulae.

**Conclusions:**

SS-OCT and PCI demonstrated good agreement on biometric measurements; however, there were significant differences in some biometric values. These differences in some ocular biometrics can cause a difference in refractive error after cataract surgery. New type SS-OCT was not superior to the IOL power prediction calculated by PCI.

## Introduction

Presently, cataract surgery is becoming an increasingly sophisticated refractive surgery as it goes beyond the treatment of vision loss caused by lens opacity. Therefore, various intraocular lens (IOL) calculation methods and biometry are developed for accurate calculation of IOL.

Recently, high-resolution swept-source optical coherence tomography (SS-OCT) has been introduced to analyze not only the posterior segments but also the anterior segment of the eyeball. It can demonstrate the structure of the eyeball, corneal topography, and biometry, including the axial length (AL), owing to the greater tissue penetration depth by the light source. SS-OCT devices currently used in clinical practice include Argos (Movu, Santa Clara, CA), IOLMaster 700 (Carl Zeiss Meditec, Jena, Germany), and OA-2000 (Tomey, Nagoya, Japan) [1–3]. The accuracy of measurements with the previously widely used IOLMaster 500 has been well-established [4–7]. The ANTERION (Heidelberg Engineering Inc., Heidelberg, Germany) is a new SS-OCT device capable of capturing a wider scan depth (14.5 mm) and scan width (16.5 mm), with a high axial resolution of < 10 μm and a light source of 1300-nm wavelength, as well as measuring the AL in the range of 14–32 mm.

There are no studies on the prediction of postoperative refractive outcomes calculated with various IOL calculation formula by the new SS-OCT ANTERION device in cataract surgery. Therefore, the present study aimed to compare the ANTERION (SS-OCT) and IOLMaster 500 (PCI) devices for measuring the major ocular biometry parameters and postoperative refractive errors.

## Material and methods

We retrospectively reviewed the medical records of patients who had undergone SS-OCT and PCI at Kim’s Eye Hospital from February 2020 to August 2020 for the analysis of their ocular biometry. The study protocol was approved by the Institutional Review Board (IRB number: 2020-12-007) at Kim’s Eye Hospital, Seoul, Korea, and the study was conducted in accordance with the tenets of the Declaration of Helsinki.

In this study, we enrolled a total of 118 eyes with a mean age of 66.43 ±0.92 years (Table 1, range: 44 to 85 years). The eyes with ophthalmologic diseases that could affect the measurement of AL, such as corneal disease, glaucoma, and retinal disease, were excluded. We also excluded those with complications that may affect refractive error measurements, such as zonulysis and rupture of the posterior lens capsule during surgery. Best-corrected visual acuity (BCVA) was measured with decimal values on the Snellen visual acuity chart. Those with BCVA < 0.8 (decimal value) 1 month postoperatively were also excluded. Patients were excluded when the AL measurements of SS-OCT and PCI were not possible owing to the lens opacities.

**Table 1:** Patient characteristics.

N = 118	Mean value ± SE	Range
**Age** (year)	66.43 ± 0.92	44 to 85
**Pre-operative MRSE (D)**	-0.83 ± 0.24	-9.25 to +3.50
**Post-operative MRSE (D)**	-0.49 ± 0.09	-4.50 to +0.75
**Pre-operative BCVA (Decimal value)**	0.55 ± 0.02	0.01 to 1.0
**Post-operative BCVA (Decimal value)**	0.96 ± 0.01	0.8 to 1.0
**IOL power (D)**	21.01 ± 0.22	12.0 to 25.5

D: Diopter.

MRSE: Manifest refraction spherical equivalent.

BCVA: Best-corrected visual acuity.

IOL: Intraocular lens.

The participants who underwent cataract surgery using clear corneal incision and posterior chamber IOL implantation with the Tecnis ZCB00 monofocal IOL (Abott Medical Optics Inc., Albuquerque, NM, USA) at Kim’s Eye Hospital were selected. All eyes underwent complete ophthalmologic examination, including preoperative and postoperative visual acuity test, noncontact tonometer, and mydriatic fundus examination. At 1 month postoperatively, the best-corrected visual acuity test (BCVA, decimal value) and manifest refraction (MR) test were performed.

### Instruments

SS-OCT measurements of the AL, anterior chamber depth (ACD) and corneal curvature were performed using the cataract application mode. The AL was defined as the distance between the anterior corneal tear film and the retinal pigment epithelium (RPE) along the line of sight. The AL calculation algorithm checks how many measurements are within 50 μm of three subset data. The ACD is defined as the distance from the anterior corneal surface to the anterior lens surface, measured perpendicular to the anterior corneal surface and along the visual axis. The corneal curvature was measured with only SS-OCT images (total of 65 radial B-scan images, 256 A-scans per B-scan) in the 3-mm zone of the central cornea. The PCI measure the AL with a 780-nm laser diode infrared light along the visual axis. The ACD was calculated the distance between light reflections on the anterior corneal surface and the anterior lens surface using by a 0.7mm-wide slit beam of light which is directed at a 30-degree angle into the anterior chamber. The anterior corneal curvature was also obtained from six hexagonal arrays reflected from the central cornea face in a plane approximately 2.3 mm in diameter.

Biometric parameters, AL, ACD, and anterior corneal curvature (K1; flat K, K2; steep K) were corrected and analyzed.

### IOL power calculation

IOL power was calculated according to the four formulas (Haigis, SRKT, Holladay1, Barrett Universal II) considering preoperative MR values, age, and lifestyle of each patient. Therefore 20 eye were targeted on myopia (-0.50D~-4.00D), 98 eye were targeted on emmetropia.

The optimized IOL (ZCB00) constants which provided by the User Group for Laser Interference Biometry (ULIB, http://ocusoft.de/ulib/c1.htm) were used for the SS-OCT and PCI (a0 = -1.302, a1 = 0.210, and a2 = 0.251 for Haigis, 119.3 for SRKT, Surgeon factor 2.02 for Holladay1 and 119.39 for Barrett Universal II). The IOLMaster 500 (ver. 5.4) does not have the Barrett Universal II formula. Therefore, the Barrett Universal II online calculator (version 1.05) from the Asia-Pacific Association of Cataract and Refractive Surgeons (https://calc.apacrs.org/barrett_universal2105/) was used. The Barrett Universal II formula is available for SS-OCT.

For calculating the prediction error of IOL power calculated with four different formulas, we assessed the difference between the measured manifest refraction spherical equivalent (MRSE) at 1 month postoperatively and the predicted refraction based on implanted IOL power. The mean arithmetic prediction error (ME), mean absolute prediction error (MAE), and percentage of eyes with a prediction error within ± 0.5 diopters (D) were analyzed [8]. The difference in the predicted refractive errors of the IOL up and down one level of the used IOL power used in the surgery was calculated based on the SRKT of PCI. 0.34 D was calculated as the average of these values and based on this value, it was investigated whether there was a difference between biometry according to each IOL calculation formula.

### Statistical analysis

Data were analyzed using SPSS, version 24.0 (IBM Corporation, Armonk, NY, USA), for Windows (Corporation, Redmond, WA, USA) and MedCalc for Windows, version 18.2.1. The Shapiro–Wilk test was used to evaluate the normality of the numerical data. A paired *t*-test and Wilcoxon signed rank test were used to compare the biometric parameters and ME and MAE between the two devices. Percentage of eyes with a prediction error within specific diopter were analyzed with Chi-square test. Pearson’s product-moment correlation coefficient was used to analyze the correlation between parameters. The Bland–Altman limit of agreement (LoA) method was used to evaluate the agreement in the biometric parameters between the two biometers [9]. Statistical significance was set at *p* < 0.05.

## Results

The mean preoperative spherical equivalent was -0.83 ± 0.24 D, and BCVA was 0.55 ± 0.02 (decimal value). The mean postoperative spherical equivalent was -0.49 ± 0.09 D, and BCVA was 0.96 ± 0.01 (decimal value) (Table 1).

**Fig 1:**
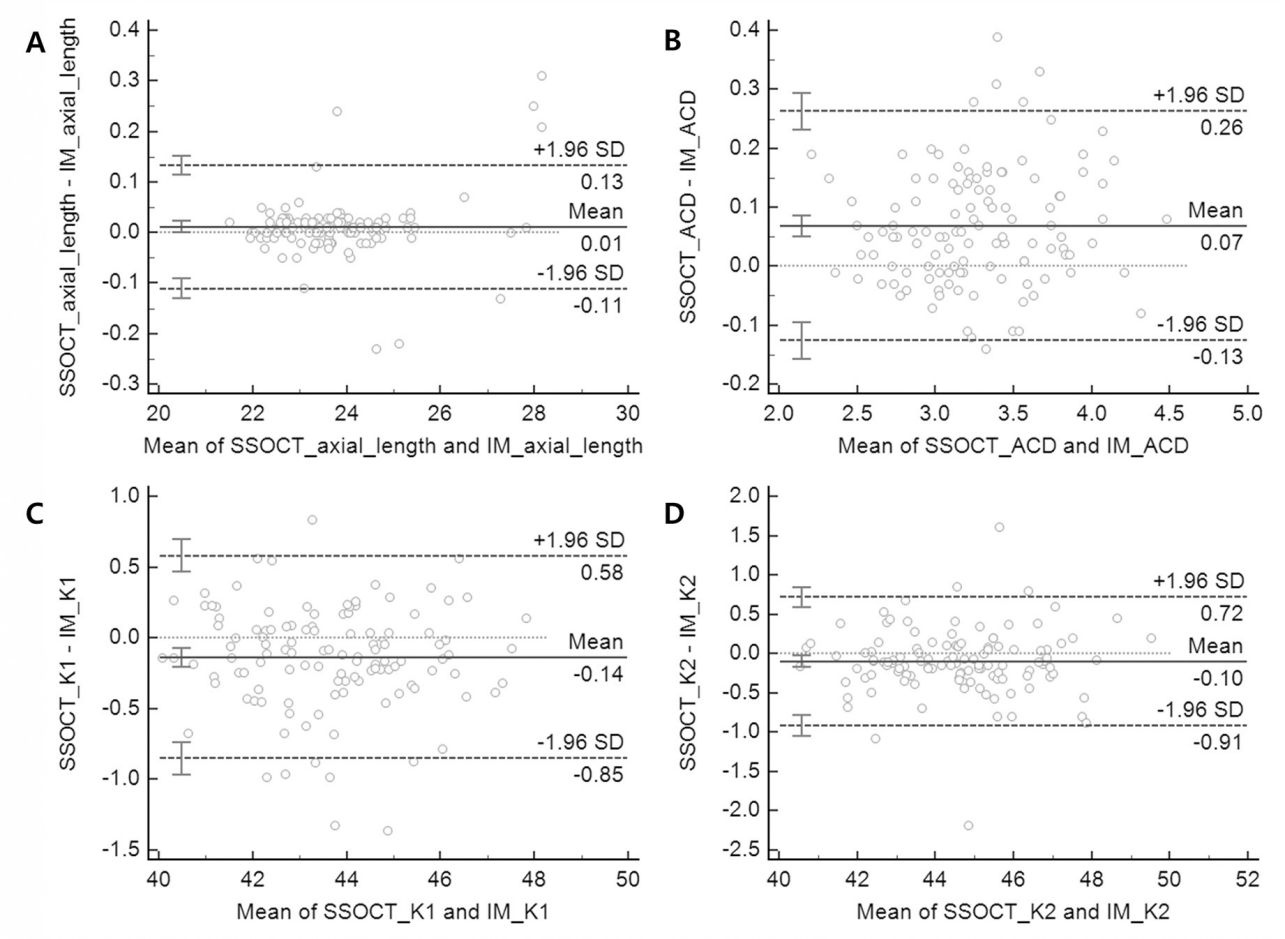
Bland-Altman plot of agreement between swept-source optical coherence tomography and partial coherence interferometry. (A) axial length, (B) ACD, (C) flat K (K1), (D) steep K (K2). The mean difference is indicated by the ㅠblack dashed lines, and 95% LoA is indicated by the black dashed line. The grey doted line indicate the draw line of equality.

The mean preoperative AL for SS-OCT and PCI were 23.78 ± 0.12 mm and 23.77 ± 0.12 mm, respectively, and demonstrated no statistically significant difference (Table 2, paired *t*-test, p > 0.05). The LoA of AL was -0.11 to 0.13, and all the measured values except those in the seven eyes, were within this range (Fig 1). The mean ACD was 3.30 ± 0.04 mm (range: 2.30 to 4.52) for SS-OCT and 3.23 ± 0.04 mm for PCI (range: 2.11 to 4.44). The mean difference was 0.07 ± 0.01, with a significant difference in the paired *t*-test (P<0.05). The LoA of ACD was -0.13 to 0.26 (Fig 1). The mean corneal curvature was as follows: SS-OCT, K1: 43.58 ± 0.16 D (range: 39.99 to 47.88), K2: 44.50 ± 0.17 D (range: 40.45 to 49.61); PCI, K1: 43.72 ± 0.16 D (range: 40.13 to 47.74), K2: 44.60 ± 0.17 D (range: 40.61 to 49.41). K1 and K2 demonstrated statistically significant differences. The mean corneal curvature difference was -0.14 ± 0.03 D in K1 and -0.10 ± 0.04 D in K2, and the LoA was -0.85 to 0.58 in K1 and -0.91 to 0.72 in K2 (Fig 1).

**Table 2:** Biometric data measured by PCI and SS-OCT †.

N = 118		Mean value ± SD (mm)	Mean arithmetic difference ± SD (mm)	Mean absolute difference ± SD (mm)	*p*-value	Pearson’s correlation	95% LoA
**AL**	SS-OCT	23.78 ± 0.12	0.01 ± 0.01	0.03 ± 0.05	>0.05	0.999 (p<0.001)	0.24(-0.11 to 0.13)
	PCI	23.77 ± 0.12					
**ACD**	SS-OCT	3.30 ± 0.04	0.07 ± 0.01	0.09 ± 0.08	<0.05	0.976 (p<0.001)	0.39(-0.13 to 0.26)
	PCI	3.23 ± 0.04					
**K1**	SS-OCT	43.58 ± 0.16	-0.14 ± 0.03	0.29 ± 0.26	<0.05	0.978 (p<0.001)	1.43(-0.85 to 0.58)
	PCI	43.72 ± 0.16					
**K2**	SS-OCT	44.50 ± 0.17	-0.10 ± 0.04	0.30 ± 0.31	<0.05	0.974 (p<0.001)	1.63(-0.91 to 0.72)
	PCI	44.60 ± 0.17					

SD, standard deviation; LoA, limits of the agreement; AL, axial length; ACD, anterior chamber depth; K1, flat corneal curvature; K2, steep corneal curvature; PCI, partial coherence interferometry; SS-OCT, swept-source optical coherence tomography.

Table 3 illustrates the comparison of target diopters to the corresponding IOL diopters calculated according to the following four formulas. The difference in target diopters calculated according to the four formulas using SS-OCT and PCI were statistically significant, except those calculated by the Barrett formula (p value>0.452).

**Table 3:** Mean arithmetic error and mean absolute error of IOL power calculation using data from two biometrics.

		SS-OCT	PCI	Mean difference	p-value
**Haigis**	**Target diopters**	-0.44 ± 0.09 (-4.15 to 0.81)	-0.55 ± 0.09 (-4.77 to 0.20)	0.11 ± 0.03	<0.001*
	**ME(D)**	-0.05 ± 0.05 (-1.70 to 1.79)	0.05 ± 0.04 (-1.16 to 1.25)	-0.11 ± 0.03	<0.001*
	**MAE(D)**	0.38 ± 0.03 (0.01 to 1.79)	0.32 ± 0.24 (0.01 to 1.25)	0.07 ± 0.02	0.006*
	**Eyes within ± 0.5D (%)**	82.20%	80.51%		0.738**
**SRKT**	**Target diopters**	-0.48 ± 0.09 (-4.43 to 0.75)	-0.53 ± 0.09 (-4.92 to 0.71)	0.05 ± 0.02	0.018*
	**ME(D)**	-0.02 ± 0.05 (-1.63 to 2.17)	0.04 ± 0.04 (-1.17 to 1.61)	-0.05 ± 0.02	0.018*
	**MAE(D)**	0.37 ± 0.03 (0.01 to 2.17)	0.32 ± 0.02 (0.01 to 1.61)	0.05 ± 0.02	0.013*
	**Eyes within ± 0.5D (%)**	77.12%	85.59%		0.095**
**Holladay1**	**Target diopters**	-0.50 ± 0.10 (-4.80 to 0.67)	-0.57 ± 0.09 (-5.40 to 0.52)	0.08 ± 0.02	0.003*
	**ME(D)**	0.01 ± 0.05 (-1.67 to 2.66)	0.08 ± 0.04 (-1.34 to 2.06)	-0.08 ± 0.02	0.003*
	**MAE(D)**	0.39 ± 0.03 (0.01 to 2.66)	0.32 ± 0.03 (0.02 to 2.06)	0.06 ± 0.02	0.005*
	**Eyes within ± 0.5D (%)**	73.73%	82.20%		0.116**
**Barrett**	**Target diopters**	-0.56 ± 0.09 (-4.34 to 0.60)	-0.54 ± 0.09 (-4.79 to 0.37)	-0.02 ± 0.03	0.452*
	**ME(D)**	0.07 ± 0.04 (-1.57 to 1.95)	0.05 ± 0.03 (-0.91 to 1.34)	0.02 ± 0.03	0.452*
	**MAE(D)**	0.34 ± 0.03 (0.01 to 1.95)	0.28 ± 0.02 (0.01 to 1.34)	0.06 ± 0.02	0.002*
	**Eyes within ± 0.5D (%)**	79.66%	84.75%		0.307**

D, diopter; ME, mean arithmetic prediction error; MAE, mean absolute prediction error; PCI, partial coherence interferometry; SS-OCT, swept-source optical coherence tomography.

* Paired T-test.

** Chi-square test.

The predictive accuracy using the four formulas is illustrated in Table 3. The ME calculated by Haigis, SRKT, Holladay1, and Barrett using SS-OCT and PCI was as follows: SS-OCT: Haigis:- 0.05 ± 0.05 D (range: -1.70 to 1.79), SRKT: -0.02 ± 0.05 D (range: -1.63 to 2.17), Holladay1: 0.01 ±0.05 D (range: -1.67 to 2.66), Barrett: 0.07 ± 0.04 D (range: -1.75 to 1.95); PCI: Haigis: 0.05 ± 0.04 D (range: -1.16 to 1.25), SRKT: 0.04 ± 0.04 D (range: -1.17 to 1.61), Holladay1: 0.08 ± 0.04 (range: -1.34 to 2.06), Barrett:0.05 ± 0.03 D (range: 0.91 to 1.34). The differences in ME measurements taken with SS-OCT and PCI were significantly different (p<0.05) among the Haigis, SRKT, and Holladay1 formulas.

The MAE was as follows: SS-OCT, Haigis: 0.38 ± 0.03 D (range: 0.01 to 1.79), SRKT: 0.37 ± 0.03 D (range: 0.01 to 2.17), Holladay1: 0.39 ± 0.03 D (range: 0.01 to 2.66), Barrett: 0.34 ± 0.03 D (range: 0.01 to 1.95); PCI: Haigis: 0.32 ± 0.24 D (range: 0.01 to 1.25), SRKT: 0.32 ± 0.02 D (range: 0.01 to 1.61), Holladay1: 0.32 ± 0.03 (range: 0.02 to 2.06), Barrett: 0.28 ± 0.02 D (range: 0.01 to 1.34). The difference in MAE measured using SS-OCT and PCI was statistically significant for all four formulas.

Table 4 shows the comparison of percentage divided by manifest refraction 0.34 D which assumed the difference by one level of IOL. There were no significant difference between two biometry, except Haigis formula (p-value = 0.035).

**Table 4:** Comparison of percentage divided by manifest refraction 0.34 D which assumed the difference by one level of IOL.

Formula	Biometry	< 0.34D	≥ 0.34D	P-value
**Haigis**	SS-OCT	51.69% (61 eyes)	48.31% (57 eyes)	0.035^*^
	PCI	65.25% (77 eyes)	34.75% (41 eyes)	
**SRKT**	SS-OCT	56.78% (67 eyes)	43.22% (51 eyes)	0.352^*^
	PCI	62.71% (74 eyes)	37.29% (44 eyes)	
**Holladay1**	SS-OCT	55.93% (66 eyes)	44.07% (52 eyes)	0.184^*^
	PCI	64.41% (76 eyes)	35.59% (42 eyes)	
**Barrett**	SS-OCT	61.86% (73 eyes)	38.14% (45 eyes)	0.274^*^
	PCI	68.64% (81 eyes)	31.36% (37 eyes)	

D, diopter; PCI, partial coherence interferometry; SS-OCT, swept-source optical coherence tomography.

* Chi-square test.

Table 5 shows the ME, MAE and percentage of eyes with a prediction error equal to or less than ± 0.5 D according to AL range. The percentages of eyes within ± 0.5 D were not significantly different among the two devices (all p value>0.05 in chi-square test). The ME, MAE also showed no significant differences among eyes with AL shorter than 22.5mm, or longer than 25mm. The difference in ME, MAE calculated using SS-OCT and PCI were statistically significant in eyes with AL between 22.5 mm and 25.0 mm, except those calculated by the Barrett formula.

**Table 5:** Comparison of postoperative refractive errors according to axial length range.

		Formula	SS-OCT	PCI	Mean difference	P-value
**AL< 22.5mm (n = 14 eyes)**	**AL (mm)**		22.18 ± 0.07	22.18 ± 0.07	0.01 ± 0.01	0.438*
	**ME (D)**	Haigis	0.12 ± 0.10	0.23 ± 0.09	-0.11 ± 0.06	0.069*
		SRKT	-0.31 ± 0.09	-0.25 ± 0.08	-0.05 ± 0.04	0.300*
		Holladay1	-0.15 ± 0.10	-0.07 ± 0.08	-0.08 ± 0.05	0.162*
		Barrett	0.01 ± 0.12	-0.01 ± 0.08	-0.01 ± 0.08	0.862*
	**MAE (D)**	Haigis	0.30 ± 0.06	0.30 ± 0.07	0.01 ± 0.04	0.552*
		SRKT	0.35 ± 0.08	0.30 ± 0.06	0.04 ± 0.04	0.490*
		Holladay1	0.31 ± 0.06	0.24 ± 0.05	0.07 ± 0.05	0.162*
		Barrett	0.34 ± 0.07	0.25 ± 0.05	0.09 ± 0.05	0.131*
	**Eyes within ± 0.5D (%)**	Haigis	85.71%	78.57%		0.622**
		SRKT	85.71%	92.86%		0.541**
		Holladay1	85.71%	85.71%		1.000**
		Barrett	71.42%	85.71%		0.357**
**22.5mm < AL < 25mm (n = 90 eyes)**	**AL (mm)**		23.69 ± 0.07	23.60 ± 0.07	0.01 ± 0.01	0.438***
	**ME (D)**	Haigis	-0.09 ± 0.05	0.03 ± 0.04	-0.13 ± 0.03	<0.001***
		SRKT	-0.02 ± 0.05	0.05 ± 0.04	-0.07 ± 0.02	0.003***
		Holladay1	-0.03 ± 0.05	0.07 ± 0.04	-0.10 ± 0.03	<0.001***
		Barrett	0.05 ± 0.05	0.05 ± 0.04	0.01 ± 0.03	0.831***
	**MAE (D)**	Haigis	0.39 ± 0.03	0.31 ± 0.03	0.09 ± 0.03	0.003***
		SRKT	0.36 ± 0.03	0.30 ± 0.03	0.06 ± 0.02	0.005***
		Holladay1	0.37 ± 0.03	0.30 ± 0.03	0.06 ± 0.02	0.009***
		Barrett	0.34 ± 0.02	0.28± 0.02	0.06 ± 0.02	0.008***
	**Eyes within ± 0.5D (%)**	Haigis	77.78%	81.11%		0.580**
		SRKT	77.78%	85.56%		0.178**
		Holladay1	73.33%	85.56%		0.064**
		Barrett	80.00%	86.67%		0.230**
**25mm < AL (n = 14 eyes)**	**AL (mm)**		26.48 ± 0.35	26.44 ± 0.33	0.04 ± 0.04	0.107*
	**ME (D)**	Haigis	-0.01 ± 0.16	-0.02 ± 0.14	0.01 ± 0.11	0.975*
		SRKT	0.30 ± 0.17	0.23 ± 0.15	0.07 ± 0.10	0.432*
		Holladay1	0.42 ± 0.22	0.34 ± 0.18	0.08 ± 0.11	0.414*
		Barrett	0.20 ± 0.15	0.09 ± 0.14	0.11 ± 0.10	0.272*
	**MAE (D)**	Haigis	0.40 ± 0.11	0.39 ± 0.09	0.01 ± 0.08	0.754*
		SRKT	0.43 ± 0.15	0.43 ± 0.11	-0.01 ± 0.09	0.925*
		Holladay1	0.60 ± 0.19	0.55 ± 0.14	0.05 ± 0.11	0.730*
		Barrett	0.37 ± 0.12	0.32 ± 0.11	0.05 ± 0.08	0.379*
	**Eyes within ± 0.5D (%)**	Haigis	85.71%	78.57%		0.622**
		SRKT	71.42%	78.57%		0.663**
		Holladay1	57.14%	57.14%		1.000**
		Barrett	92.86%	78.57%		0.280**

AL, axial length; D, diopter; ME, mean arithmetic prediction error; MAE, mean absolute prediction error; PCI, partial coherence interferometry; SS-OCT, swept-source optical coherence tomography.

* Wilcoxon signed rank test.

** Chi-square test.

***Paired T-test.

**Fig 2:**
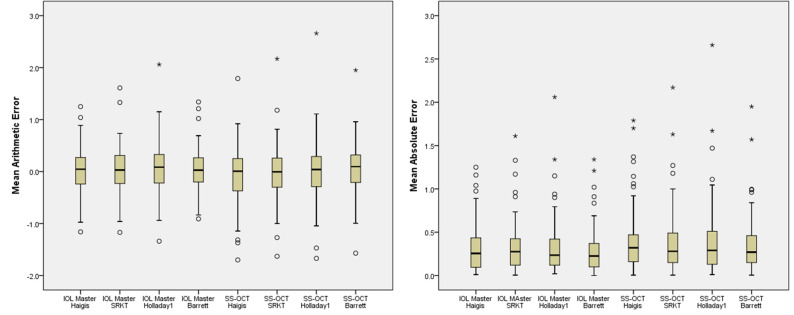
Box plot of mean arithmetic error and mean absolute error of IOL power calculated with four formulas. Round mark (o) means outlier values which are between one and a half and three box lengths; asterisk(*) means extreme values which are more than three box lengths.

Fig 2 illustrates the box plots and distributions of ME and MAE of IOL power calculated with four formulas. The formula with the least outliers of ME were the Haigis formula for PCI the Barrett Univerisial II formula for SS-OCT. The interesting finding is the extreme far outliers were showed in Holladay1 formula for both biometries.

**Fig 3:**
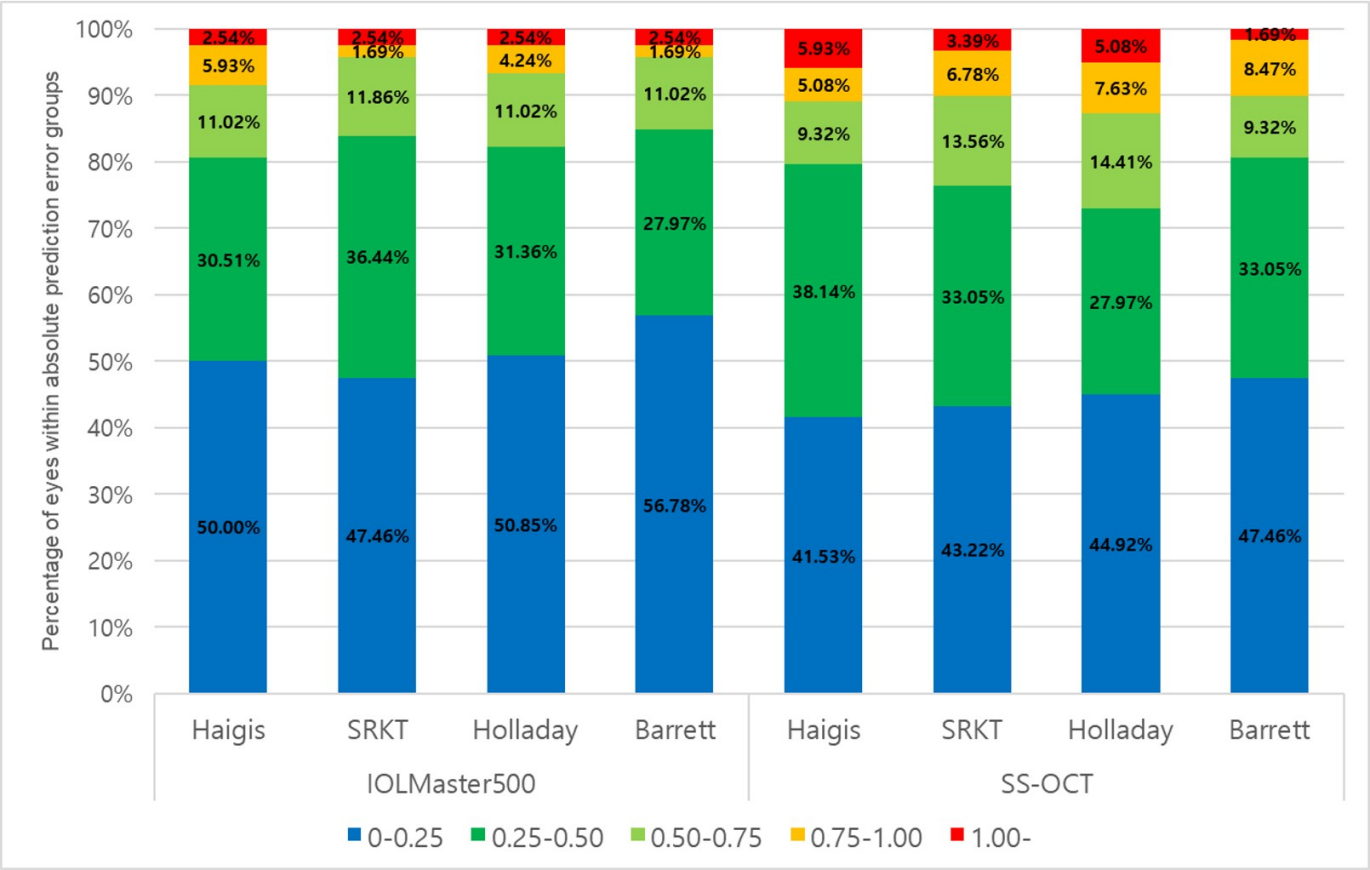
Stacked graph represents the percentage of eyes achieved the absolute prediction errors by four formulas with SS-OCT and PCI.

The MAE was ± 0.50 D or less in at least 80% of eyes with all formulas for PCI, but with only Barrett Universial II formula for SS-OCT (Fig 3). The highest percentages were achieved with the Barrett Universial II (84.75%), followed by the SRKT (83.91%), Holladay1 (82.21%) and Haigis (80.51%) for PCI. The highest percentages were achieved with the Barrett Universial II (80.51%), followed by the Haigis (79.67%), SRKT (76.27%) and Holladay1 (72.89%) for SS-OCT.

## Discussion

Among SS-OCT devices currently used in clinical practice, Argos, IOL Master 700, and OA-2000 have been previously studied [1–3]. Fișuș AD et al. demonstrated that the ANTERION had a good correlation and agreement with IOLMaster 700 for all the biometric parameters including AL, keratometry, ACD, lens thickness, and central corneal thickness [10]. Ramón et al. demonstrated that ANTERION had good repeatability for different ocular biometric measurements [11].

In this study, we compared the prediction of postoperative refractive outcomes of four different formulas (Haigis, SRKT, Holladay1, and Barrett Universal II) measured by SS-OCT and PCI. To the best of our knowledge, this is the first study to compare the refraction errors after cataract surgery measured by SS-OCT (ANTERION) and PCI (IOLMaster 500). Currently, PCI is considered the standard method for ocular biometry [12]. PCI has good repeatability and accuracy of AL assessment, and many cataract surgeries are performed using PCI-based biometry [4–7].

In this study, the AL measurement value did not show a statistically significant difference on comparing PCI (23.77 ± 0.12 mm) with SS-OCT (23.78 ± 0.12 mm) (Table 2, paired t-test, P>0.05). The mean difference in AL between SS-OCT and PCI was 0.01 ± 0.01, which was very small. In general, these differences between the two biometry devices would not be clinically significant because a 0.00~0.05 mm difference in the AL would result in a less than 0.1 D difference in postoperative refractive errors [13]. In a previous study, Higashiyama et al. compared ARGOS SS-OCT and IOLMaster 500 and noted no significant difference in the AL measurement [14]. An Y et al. expected significant differences in the measured values of the AL between ARGOS SS-OCT and IOLMaster 500 owing to different measurement principles; however, there was no statistical significance in patients undergoing cataract surgery (ARGOS: 24.56 ± 2.16 mm; IOLMaster 500: 24.58 ± 2.22 mm, *p*>0.05) [15]. In summary, the similarity between the previous study and our study is that the measurement of AL using SS-OCT and PCI will not show significant clinical differences after cataract surgery. The measurements of AL using SS-OCT could be more effective in obtaining biometric measurements in eyes with posterior subcapsular and dense nuclear cataracts owing to deeper tissue penetration [2].

SS-OCT biometry devices provide a more precise ACD measurement since the measurements are acquired along the optical axis, independent of the subject’s fixation angle [16]. In previous study, the mean ACD (central cornea thickness + anterior aqueous depth) measured by SS-OCT were 3.20 ± 0.42 mm [10], 3.45 ± 0.54 mm [17] and the mean anterior aqueous depth (AQD) were 2.89 ± 0.70 mm [18], 2.82 ± 0.49 mm [19]. In our study, ACD also showed a statistically significant difference between SS-OCT (3.30 ± 0.04 mm) and PCI (3.23 ±0.04 mm). ACD was deeper when measured by SS-OCT than by PCI. The mean difference was 0.07 ± 0.01. IOLMaster 500 uses the principle of PCI for AL measurement; however, the ACD is measured by optical principles using a non-PCI method. SS-OCT is measured by auto segmentation of ACD by directly tomographic images of the cornea and lens based on the optical axis. We believe that this difference in the measurement principle may cause a difference in the ACD value. SS-OCT can measure more precise anterior segment tomographic images since the ability to use longer wavelengths of SS-OCT than those used by PCI (780 nm) reduces light scattering [20].

In this study, corneal curvature values demonstrated statistically significant differences in both K1 and K2 values. Both K1 and K2 showed smaller values in SS-OCT than in PCI. The difference in corneal curvature is assumed to be owing to differences in measurement methods. PCI uses a distance-independent telecentric keratometry system, which measures the curvature of the light source by projecting it to the cornea. PCI measures the corneal curvature at six hexagonal points in a central 2.3 mm area, and SS-OCT measures the simulated anterior corneal curvature in a 3 mm zone with a 65-radial scan, which is assumed to make a difference in the average corneal curvature measurement. It is thought that SS-OCT measures a larger number of scans over a wide range compared to PCI.

For calculating the prediction error of IOL power calculated with four different formulas, the ME values tend to be smaller in SS-OCT than in PCI except when calculated using the Barrett formula. This means that there is a tendency for more myopic changes after surgery when using SS-OCT. SS-OCT devices showed slightly more hyperopic tendency than the preoperative target refraction. When comparing postoperative MAE, there were statistically significant differences for each formula (Table 3). MAE values tended to be larger in SS-OCT than in PCI. The MAE values are the smallest when using the Barrett formula in both SS-OCT and PCI (0.34 ± 0.03 D, 0.28 ± 002 D, respectively). The smallest mean difference in MAE between SS-OCT and PCI was SRKT (0.05 ± 0.02). Most of the mean differences are less than 0.1 diopters, it is thought that there is no clinically meaningful difference between the two devices. When evaluate which formula would lead to a different IOL choice (at least 0.5D on IOL level) (Table 4), the significant difference was only seen in the haigis formula. In the Haigis formula, the difference in ME between the two devices was also the largest, around -0.11 ± 0.03 D, so when calculated using the Haigis formula, it suggests that there may be an IOL calculation error compared to other IOL calculation formula.

In the comparison divided by the AL, the ME and MAE results were shown in a similar pattern to that of all subjects of the 22.5–25.0mm AL group. However, in the small (AL<22.5mm) and large eyes (AL>25.0 mm), there was no significant difference between the two devices. This may be a selective bias according to the difference in study sample number, so it is thought that comparative analysis is necessary for a larger number. The percentage of eyes within ± 0.5 D were no statistically significant difference among the two devices.

There can be some reasons for these refractive outcomes. First, these results may be due to the differences in corneal curvature measurements between the two biometers. Since corneal curvature is used as a variable in all formulas used above, the difference in corneal curvature will be the cause of the predictive power difference between the two devices in all formulas. The difference in variables using more flatten axial corneal curvature for SS-OCT may be a factor in the different IOL power calculation. SS-OCT measures axial corneal curvature flatter than PCI because it measures a larger number of B-scans in a wide range. Although the same refractive index (1.3375) was applied, the difference in the imaging area and the difference in the light source will cause corneal curvature. Hence, when using the same IOL diopter, a relatively myopic tendency may be observed in the SS-OCT results (Table 3). In a previous study comparing other SS-OCT (CASIA2 and IOLMaster 700), the corneal curvature was measured relatively small in ANTERION [21]. In this study, the corneal power difference of about 0.1 D is about 0.14 D in the IOL plane, assuming the IOL plane to the corneal plane equivalent to the power conversion factor is about 0.69 [22]. However, with a difference of less than 0.34D, this is not enough to cause a difference in the IOL power step.

This difference in ACD can be a factor of error in IOL calculations using effective lens position (ELP) as a major variable, such as Haigis formula, and may cause a change in refractive power after cataract surgery in shallow ACD or high myopia [23]. The ACD measured with PCI was smaller than SS-OCT in this study (mean difference = 0.07 ± 0.01). Therefore, the ELP after surgery is applied to the Haigis formula as being more anteriorly with PCI. This may be the reason that the predicted refractive power calculated by PCI has a myopic value than calculated by SS-OCT in Haigis formula, and when the target refractive power is set as the emmetropia, the postoperative refractive error shows a relatively hyperopic change. Even though the ACD measurement also shows a statistically significant difference, the mean difference value is too small to determine the IOL diopter; hence, it is not clinically significant. Of the four formulas we used, the Haigis formula and the Barrett Universal II formula used ACD as a variable, but the Barrett Universal II did not show a significantly different ME. Finally, the mean difference in the AL value was not only small but was also not significant; it is unlikely to affect the refraction error difference between the SS-OCT and PCI.

Each formula has a difference in MAE because of the difference in the variables. In the Haigis formula and Barrett universal II formula, the ACD is included in the variable and is not included in SRKT and Holladay1 [24]. Unlike in PCI, if the lens thickness or WTW measured in SS-OCT is entered as a variable in the Barrett universal II formula, a more accurate prediction error can be produced. Despite the statistical significance of MAE, it may have little clinical significance since the mean difference is too small and within 0.1 D. Since refractive errors after cataract surgery always exist for each biometry and for each IOL calculation formula in calculating the IOL power, the best thing is to select the IOL diopter that is expected to have the least error through comparison of several formulas. Therefore, it is meaningful to compare and analyze the refractive errors of various devices and calculations.

In a previous study, Cheng et al. reported that the most accurate prediction of postoperative refraction can be achieved with the Barrett formula when IOLMaster 700 was used for biometric measurement [25]. Kane et al. reported that Barrett Universal II produced the most accurate outcome in the medium AL group when PCI biometry was used [26]. In our study, as in the previous study, the Barrett Universal II formula with the PCI biometer yielded the highest predictive accuracy. However, when using Barrett Universal II also showed the lowest MAE value in SS-OCT (0.34 ± 0.03) and PCI (0.28 ± 0.02), and the percentage of eyes with a prediction error equal to or less than ± 0.50 D also showed the highest value in both SS-OCT and PCI. Interestingly, in the SS-OCT, the percentage of eyes with a prediction error equal to or less than ± 0.50 D was about 80% only in the Barrett Universal II formula. Rather, in the PCI, it was over 80% in all formulas. The IOL constant values for each formula used in this study were an optimized constant provided by the ULIB calculated by optical biometry in many previous studies. If the personalized IOL constant value calculated by SS-OCT was applied individually to each formula, it would be possible to predict more accurate IOL power even in SS-OCT. Further studies on the optimization of IOL constants for various IOL and IOL calculation formulas using SS-OCT will be needed.

The limitation of this study is its retrospective design and the small number of samples. This study does not evaluate many eyes with AL longer than 25 mm or shorter than 22 mm. It is necessary to confirm the difference of AL measurement and the change in refractive outcome according to measurements in long eyes and short eyes with larger sample sizes. In addition, it was not studied by dividing by various corneal curvatures or ACD. However, we can see general clinical trends in our study. Another limitation is that all subjects are Asian. Therefore, the preoperative power was generally myopia, and the target power was not all emmetropia. Our results may not be generalizable to other ethnicities.

In summary, biometry measured using SS-OCT showed statistically significant differences except for AL, although statistically significant, the biometric value itself is not clinically relevant since the mean difference was too small and the correlation was significant in this study. This study demonstrated a comparative refractive error between SS-OCT and PCI for biometry in cataract surgery. New type SS-OCT was not superior to the IOL power prediction calculated by PCI. Although differences were found to be small, the parameters measured by the two biometrics should not be used interchangeably. As the IOL calculations used have been calibrated through many PCI-based previous studies, further research will be needed to make more accurate prediction of IOL calculation through processes such as calibrating biometric parameters or optimizing the IOL constant by SS-OCT.

## Supporting information

S1 File Data analyzed. (XLSX)
